# Intranasal Oxytocin Treatment Increases Eye-Gaze Behavior toward the Owner in Ancient Japanese Dog Breeds

**DOI:** 10.3389/fpsyg.2017.01624

**Published:** 2017-09-21

**Authors:** Miho Nagasawa, Misato Ogawa, Kazutaka Mogi, Takefumi Kikusui

**Affiliations:** Companion Animal Research, School of Veterinary Medicine, Azabu University Sagamihara, Japan

**Keywords:** human-dog bond, oxytocin, gaze, positive loop, Japanese breeds, heart rate variability

## Abstract

Dogs acquired unique cognitive abilities during domestication, which is thought to have contributed to the formation of the human-dog bond. In European breeds, but not in wolves, a dog’s gazing behavior plays an important role in affiliative interactions with humans and stimulates oxytocin secretion in both humans and dogs, which suggests that this interspecies oxytocin and gaze-mediated bonding was also acquired during domestication. In this study, we investigated whether Japanese breeds, which are classified as ancient breeds and are relatively close to wolves genetically, establish a bond with their owners through gazing behavior. The subject dogs were treated with either oxytocin or saline before the starting of the behavioral testing. We also evaluated physiological changes in the owners during mutual gazing by analyzing their heart rate variability (HRV) and subsequent urinary oxytocin levels in both dogs and their owners. We found that oxytocin treatment enhanced the gazing behavior of Japanese dogs and increased their owners’ urinary oxytocin levels, as was seen with European breeds; however, the measured durations of skin contact and proximity to their owners were relatively low. In the owners’ HRV readings, inter-beat (R-R) intervals (RRI), the standard deviation of normal to normal inter-beat (R-R) intervals (SDNN), and the root mean square of successive heartbeat interval differences (RMSSD) were lower when the dogs were treated with oxytocin compared with saline. Furthermore, the owners of female dogs showed lower SDNN than the owners of male dogs. These results suggest that the owners of female Japanese dogs exhibit more tension during interactions, and apart from gazing behavior, the dogs may show sex differences in their interactions with humans as well. They also suggest that Japanese dogs use eye-gazing as an attachment behavior toward humans similar to European breeds; however, there is a disparity between the dog sexes when it comes to the owners’ oxytocin secretion. Japanese dogs also showed different attachment behaviors from both European breeds and wolves, and they likely use additional strategies to substitute gaze when forming the human–dog bond.

## Introduction

The dog (*Canis familiaris*) was the first animal to be domesticated (Serpell, 2016), with hundreds of different dog breeds recognized today. During the domestication process, dogs were subjected to a strong selection according to their temperament, behavior, and cognitive abilities (Hare and Tomasello, 2005). Dogs are skilled at understanding human communicative gestures compared with wolves and chimpanzees (Hare et al., 2002). They even look back at humans when encountering unsolvable tasks, while wolves do not (Miklósi et al., 2003). These findings suggest that dogs acquired their unique cognitive abilities during domestication.

The shared communicative signals with humans, such as eye-gaze, might also be related to interspecies’ bonding, namely human–dog bonding. Dogs can distinguish between individual humans (Nagasawa et al., 2009) and show distinctly different behaviors to caregivers compared with hand-raised wolves (Topál et al., 2005). Furthermore, interaction with dogs confers a social buffering effect to humans (Polheber and Matchock, 2014). Likewise, dogs also receive more social buffering effects from interacting with humans than from conspecifics (Tuber et al., 1996). Nagasawa et al. (2009, 2015) hypothesized that an oxytocin-mediated positive loop exists between humans and dogs, which is mediated by gazing, an attachment behavior observed in human mothers and infants. Indeed, they reported that dog gazing behavior to the owner, which was not observed in wolves, increased urinary oxytocin concentrations in owners, which consequently facilitated owners’ affiliation and increased urinary oxytocin concentrations in dogs. Furthermore, nasally administered oxytocin increased the gazing behavior in dogs, which in turn increased urinary oxytocin concentrations in owners. These results show the existence of an oxytocin-mediated positive loop in human-dog relationships similar to that of human mother–infant relations (Feldman, 2012), and support that dogs might have acquired this interspecies bonding feature by the domestication process. The relationship between oxytocin and human-directed social behavior in dogs has recently become clearer. Kis et al. (2014) showed that the polymorphisms of the oxytocin receptor gene are related to social behavior such as proximity seeking and friendliness toward humans. Kovács et al. (2016) administrated oxytocin to different types of working dog breeds (Border Collie and Siberian Husky, the former is the cooperative-working type and the latter is the independent-working type) and compared their social behavior toward humans. They found that Border Collies looked more at their owners and the experimenter than Siberian Huskies after oxytocin administration. These studies indicated that the oxytocinergic system modulates dog social behavior as well as that of humans.

Recently, genetic characteristics among dog breeds have been studied and their genetic classifications constructed. A cladogram analysis of dog genes revealed a unique clade between European-originating breeds and wolves which was categorized as ‘ancient breeds.’ This category includes popular Japanese breeds such as Shiba and Akita, and a large group of breeds with presumed modern European origins (Parker et al., 2004; vonholdt et al., 2010). Since these ancient breeds are most closely related to wolves genetically, sharing the most recent common ancestor, they may show different behavioral characteristics when compared with other breed groups. Ito et al. (2004) identified a polymorphism region in the dopamine receptor D4 gene in canine breeds which was associated with human-directed aggressive traits. Moreover, through a neighbor-joining tree based on allele frequencies, breeds were divided into two main groups, whereby the group including Japanese breeds showed the highest scores in human-directed aggressive traits, assessed by a questionnaire administered to dog specialists, when compared with breeds of Occidental origin. The Japanese breed, Shiba Inu, showed the most pronounced human-directed aggression (Arata et al., 2014). Moreover, Tonoike et al. (2015) investigated whether dogs’ behavioral characteristics were different among genetically clustered breed groups and found that the dogs in the ancient and spitz breed groups showed low attachment and attention-seeking behaviors to their owners. It was also found that it took longer for ancient breeds to make eye contact with humans, and they gazed at humans for shorter periods compared with other breed groups during an unsolvable situation (Konno et al., 2016). These characteristics in Japanese breeds may stem from the fact that Japanese breeds were not selected for a particular cooperative function, so they are not highly domesticated and are genetically close to wolves.

Based on these previous studies, the ancient breeds were expected to display less attachment behavior more similar to wolves when compared with European breeds. Only limited studies have examined the effects of exogenous oxytocin in ancient breeds (Kovács et al., 2016), and revealed that the responses to oxytocin were different from a European breed; Border Collies gazed more at humans than Siberian Huskies after oxytocin administration. While some reports demonstrated that human-directed behavior is modulated by experience and learning to some degree (Barber et al., 2016; D’Aniello and Scandurra, 2016), there should be genetic modulation in these behaviors as well (Nagasawa et al., 2015). Therefore, we hypothesized that Japanese dogs show intermediate bonding traits between those of wolves and European dog breeds; therefore we tested the existence of an oxytocin-mediated positive loop in ancient breeds, Japanese dogs. We investigated whether Japanese breeds showed the oxytocin-gaze bonding system with their owner using the same procedure as Nagasawa et al. (2015). We predicted that oxytocin would increase a dog’s gazing behavior while simultaneously increasing the owner’s urinary oxytocin levels, to a lower degree than those seen in European breeds. We also attached heart rate monitors to both dogs and owners in order to monitor physiological changes during the interaction. Interaction with dogs has been shown to be effective in reducing human stress (Odendaal and Meintjes, 2003; Nagasawa et al., 2015). Emotional status, including stress and tension, can be assessed by the balance between the sympathetic and parasympathetic nervous systems, and heart rate variability (HRV) is one of the most widely used metrics of this balance. There are many methods to evaluate HRV, and the time domain methods are the simplest ones (Task Force of The European Society of Cardiology and The North American Society of Pacing and Electrophysiology, 1996). Although there’s room for consideration, some studies showed the combination of parameters analyzed in the time domain methods could indicate the emotional states (human: Kreibig, 2010, dog: Gácsi et al., 2013; Katayama et al., 2016). It was also demonstrated that oxytocin can increase HRV (Norman et al., 2011; Kemp et al., 2012). Increase of oxytocin in humans also facilitated females’ reactivity to infant crying (Riem et al., 2011). Infant crying is one of the attachment/alarming signals from infants to mothers, suggesting that attachment signals can increase tension/arousal in mothers. Therefore, if oxytocin administration increases dogs’ gazing behavior toward their owners and stimulates oxytocin secretion in the human, we have the following two hypotheses; one is that mutual gazing will lead to an activation of the parasympathetic nervous system in owners, which would be reflected in a change in the HRV, and the other is that changes of the dog’s behavior also lead to owners’ emotional arousal or tension, thereby, activates the sympathetic nervous system.

## Materials and Methods

### Participants

This experiment involved 21 volunteers (male: *n* = 11; female: *n* = 10) and their 22 dogs (5 gonadally intact male, 5 castrated, 4 gonadally intact female, 8 spayed; age: male 5.2 ± 1.0 years, female 4.3 ± 1.0 years; 18 Shiba, 2 Kai, and 2 Shikoku). Participants were recruited in dog training classes, veterinary clinics, dog runs, and through the internet. Written informed consent was obtained from the owners. We also recruited students and staff from the university who matched the owners in sex and appearance, but were unfamiliar to the dogs, to serve as unfamiliar people during the 30-min interaction. Owners and the unfamiliar people were aware of the procedure of the sessions, but blinded to its purpose and treatment. We conducted two experimental sessions, under conditions of oxytocin and saline treatment, per a dog on different days, at least 1 month apart. The order of treatment was counterbalanced.

### Procedures

Experiments were conducted in an area (4.5 × 4.5 m) that was divided by partitions in a room at Azabu University. Three chairs were set in a circle in the experimental area, and marks were placed at a 0.7-m radius from each chair with vinyl tape. We attached heart rate monitors to the chests of both owner and dog to measure HRV. If a dog reacted adversely to wearing a heart rate monitor, it was exempted from the HRV measurement. This experiment consisted of three phases: resting before the interaction, a 30-min interaction between the participants and dog, and resting after the interaction. Two video cameras (GZ-HM670, Victor, Japan) were placed in the corners, and two others (PC-355micro, Sun-Mechatronics, Japan) were placed on the ceiling of the experimental room to record the behaviors of the dogs during the 30-min interaction. The owner and his/her dog avoided eating and drinking other than water from 2 h before the experiment as well as during the experiment. The dog urinated on the way to the experimental room after arrival at the university by a car. The owner also urinated 1 h before the 30-min interaction to empty the bladder, then rested on one of the chairs with the dog, but without interacting. Immediately prior to the 30-min interaction phase, the experimenter took out the dog to urinate and the dog’s urine sample was collected by the experimenter using absorbent cotton. At the same time, the owner collected his/her own urine using a paper cup in the restroom and placed the sample in a cold reserving box. The experimenter removed the sample from the cold reserving box as soon as the owner left the restroom. Urine samples of both dog and owner were centrifuged at 4°C in a refrigerated centrifuge, and the supernatants were frozen at -80°C until assay. While the owner and the dog were providing urine samples, two unfamiliar people entered the experimental area and sat on the chairs. The order of seating was randomized by using the randomize function in Excel (Microsoft).

After the owner was seated, 100 μL of oxytocin (40 IU) or 100 μL of saline was intra-nasally administered to the dog by a hand-compressing air spray bottle. Following oxytocin or saline administration, the dog entered the experimental area. All participants were instructed to remain seated in their chairs, while the dog was allowed to move freely in the room. In order to prompt the dog’s movement during the interaction, participants were instructed to change their seats every 10 min. They were not allowed to talk to each other or to talk to and touch the dog voluntarily; however, if the dog touched participants, the participants were allowed to pet the dog in return. After the 30-min interaction, the unfamiliar people left the experimental area. The owner and the dog remained in the room and rested. Subsequently, a second urine sample was collected from both dog and owner 30 min after the interaction. Ethical approval for this study was provided by the Ethics Committee of Azabu University (#131119-1), which follows “Guidelines for Proper Conduct of Animal Experiments” by Science Council of Japan (2006).

### Outcome Measurements

#### Dog Behavioral Assessment

The dog’s behaviors were recorded using video cameras during the 30-min interaction. We measured the total amount of time during which the dog touched the participants (*touch*), was at close proximity to participants (*proximity*; dog’s head or body was within a 0.7-m radius from a participant), and has its nose oriented toward the participant’s face (*gaze*). These videos were analyzed by two persons who were blinded to the details of the study. The scores obtained by both observers were highly correlated (rs = 0.92–1.00, *p* < 0.01). Additionally using stopwatches, the participants recorded the total amount of time during which they met the dog’s eyes (“mutual gaze,” which was defined as the orientation of the dog’s nose toward the participant’s face or when the dog’s eyelids and eyebrows lifted to see the participant’s face). These scores significantly correlated with the *gaze* duration (rs = 0.88, *p* < 0.01). The average of each behavioral variable recorded by the two unfamiliar persons was used in the analysis.

#### Measurement of Urinary Oxytocin in Owners and Dogs

Immediately after collection, urine samples were frozen at -80°C until the assay was performed. After thawing, urine samples were centrifuged at 4°C, and urinary oxytocin concentrations were measured by radioimmunoassay (Mitsui et al., 2011). Urinary oxytocin concentrations were corrected by using creatinine concentrations.

#### Heart Rate Variability

Heart rate variability was telemetrically measured using a Polar RS800CX digital system device attached to the chests of owners and dogs during the experiment. The dogs that gazed at their owner for less than 5 s or reacted adversely to wearing a heart rate monitor and the owners whose data included missing values were excluded from the analysis. We selected 15-s periods before and after the period during which the dog and its owner gazed mutually in a state of calmness using Kubios Heart Rate Variability Analysis Software 2.0 for Windows (Kubios Ltd., Finland). The heart rate during these periods was converted to inter-beat (R-R) intervals (RRI). Subsequently, we calculated the following HRV parameters: (1) the standard deviation of normal to normal R-R intervals (SDNN), which is an index of the autonomic nervous system and (2) the root mean square of successive heartbeat interval differences (RMSSD), which is an index of the parasympathetic nervous system. We compared between these parameters 15 s before (pre) and after (post) mutual-gaze under two administration conditions, oxytocin and saline. It was difficult to obtain sufficient data per dog from one period; therefore, we collected two periods for each dog and owner, and combined them together in the analysis.

### Statistical Analysis

Most variables were not distributed normally; therefore, after logarithmic conversion, a linear mixed model (LMM) was conducted to compare behaviors using the factors “dog’s sex,” “participant” (owner and unfamiliar people), and “administration” (oxytocin and saline), and the dog’s identity as a random factor for each behavioral variable. Urinary oxytocin concentrations and the parameters of HRV for both dogs and their owners were also analyzed by a LMM using “dog’s sex,” “administration,” and “time of data collection” (pre and post) as factors, and the dog’s identity as a random factor, after logarithmic conversion. If significant differences were observed, a *post hoc* analysis with a Bonferroni correction was conducted. The relationships between the variables were examined using a multiple linear regression analysis. The objective variable was the oxytocin change ratio, which was calculated by dividing the post-interaction urinary oxytocin level by that of pre-interaction, and the explanatory variables were dog’s sex (male dog = 0, female dog = 1), owner’s sex (male = 0, female = 1), administration (saline = 0, oxytocin = 1), duration of *touch*, duration of *proximity*, and duration of *gaze*. Results were expressed as mean ± standard error of the mean (SE). Statistical significance was considered at *p* < 0.05. All statistical analyses were performed using SPSS software v.24.0 (IBM Japan, Tokyo).

## Results

### Differences in Behavioral Variables between the Two Treatment Groups

**FIGURE 1:**
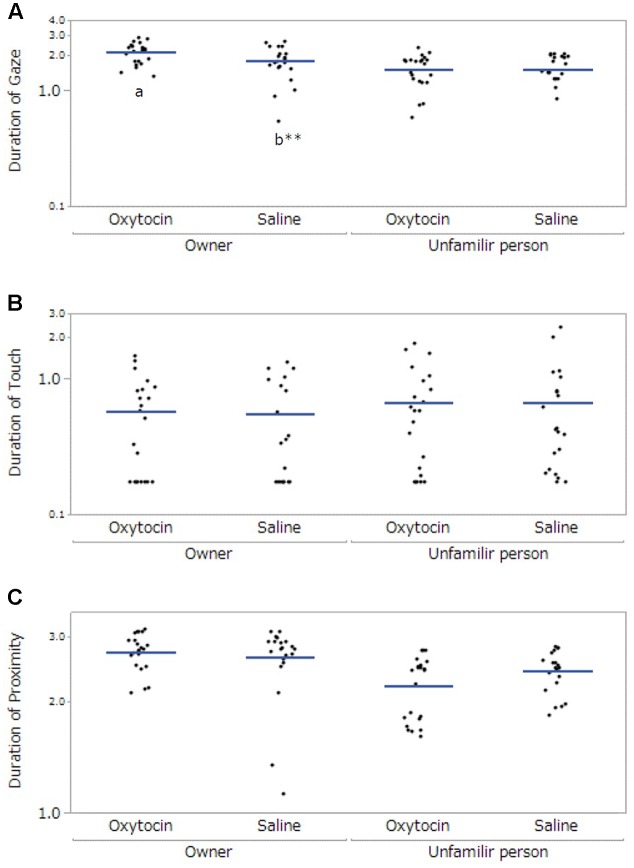
Comparisons of the dogs’ behaviors between oxytocin and saline treatment conditions. Panels show the mean duration of time of dogs’ gaze at participants **(A)**, touching participants **(B)**, and at proximity of less than 0.7 m from each participant **(C)**. A significant difference between treatment conditions was observed only in gazing behavior. Results are shown with logarithmic. The blue lines indicates the mean duration of behaviors. ^∗∗^*p* < 0.001.

We observed the following significant main effects and interactions. For *gaze*, there were significant main effects of participant (*F*[1,57.99] = 29.84, *p* < 0.001) and administration (*F*[1,58.92] = 4.13, *p* = 0.047), and a significant interaction between participant and administration (*F*[1,57.99] = 5.55, *p* = 0.02). The *post hoc* tests with a Bonferroni correction showed that dogs exhibited gazing for a significantly greater amount of time toward their owners than toward unfamiliar people in both administration conditions (oxytocin administration: *p* < 0.001, saline: *p* = 0.035). Dogs also gazed at their owners significantly longer following oxytocin administration than following saline administration (*p* = 0.003, **Figure 1A**). For *touch*, there were no significant differences (dog’s sex: *F*[1,20.09] = 2.45, *p* = 0.13, participant: *F*[1,58.13] = 1.02, *p* = 0.32, administration: *F*[1,59.38] = 0.12, *p* = 0.73, **Figure 1B**). Finally, for *proximity*, there was a significant main effect of participant (*F*[1, 58.44] = 17.23, *p* < 0.001). The *post hoc* tests with a Bonferroni correction showed that dogs kept proximity longer to their owners than unfamiliar persons (*p* < 0.001); however, we did not found the significant difference between oxytocin and saline administrations (**Figure 1C**).

### Differences in Urinary Oxytocin Concentrations between the Two Treatments

**FIGURE 2:**
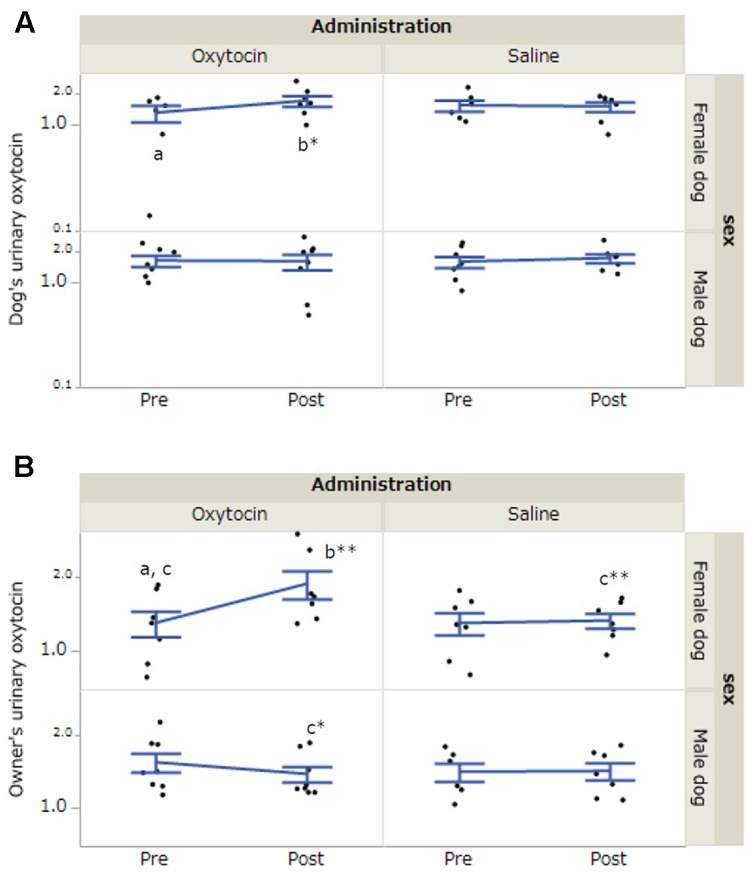
Comparisons of urinary oxytocin concentrations between oxytocin and saline treatment conditions. **(A,B)** Significant differences depending on treatment conditions were found in both female dogs and their owners. Results are shown as mean ± SE with logarithmic. ^∗^*p* < 0.05, ^∗∗^*p* < 0.001.

In dogs, there was a significant interaction between dog’s sex, administration, and time of urine collection (*F*[1,36.16] = 5.80, *p* = 0.02). A *post hoc* test with a Bonferroni correction indicated a significant increase in urinary oxytocin concentrations in female dogs after 30-min interaction in the oxytocin condition (*p* = 0.014, **Figure 2A**). In the owners, the results of LLM showed significant interactions between dog’s sex and time of urine collection (*F*[1,36.17] = 7.18, *p* = 0.01) and dog’s sex, administration, and time of urine collection (*F*[1,36.17] = 6.03, *p* = 0.02). A *post hoc* test with a Bonferroni correction indicated a significant increase in urinary oxytocin concentrations in the owners of female dogs after 30-min interaction in the oxytocin condition (*p* < 0.001). Post-interaction urinary oxytocin concentrations in the owners of female dogs were significantly higher in the oxytocin condition relative to the saline condition (*p* = 0.001). Furthermore, post-interaction urinary oxytocin concentrations were significantly higher in the owners of female dogs than in the owners of male dogs in the oxytocin treatment group (*p* = 0.02, **Figure 2B**). No significant differences were observed when we included the factor of “owner’s sex” in LLM instead of “dog’s sex.” The intra-assay coefficient of variation (CV) of the oxytocin assay corresponded to 4.05%.

### Multiple Regression of Urinary Oxytocin Concentrations in Owners and Dogs

To analyze the effect of dogs’ behaviors on urinary oxytocin concentrations in owners and dogs, a multiple linear regression analysis was conducted to explain the oxytocin change ratio in owners using the following factors: dog’s sex (male dog = 0, female dog = 1), owner’s sex (male = 0, female = 1), administration (saline = 0, oxytocin = 1), duration of *touch*, duration of *proximity*, and duration of *gaze*. Results indicated that the oxytocin change ratio in owners showed a significant regression equation (Adjusted *R*^2^ = 0.304, *F*[6, 21] = 2.969, *p* = 0.029). The dog’s sex and the duration of *touch* were significantly related to the oxytocin change ratio in owners (dog’s sex: β = 0.510, *p* = 0.011, *touch*: β = -0.490, *p* = 0.019, the oxytocin change ratio in owners = -0.055 + 0.300 ^∗^ dog’s sex + 0.136 ^∗^ administration + 0.072 ^∗^ *gaze* + (-0.375) ^∗^ *touch* + 0.145 ^∗^ *proximity* + 0.159 ^∗^ owner’s sex). That is, the owners of female dogs showed a higher increase in oxytocin, and the longer the duration of touch, the lower the increase ratio of oxytocin. In dogs, no significant regression equation was observed (Adjusted *R*^2^ = 0.133, *F*[6,19] = 0.509, *p* = 0.794).

### The Heart Rate Variability in Owners during the Interaction with Their Dogs

**FIGURE 3:**
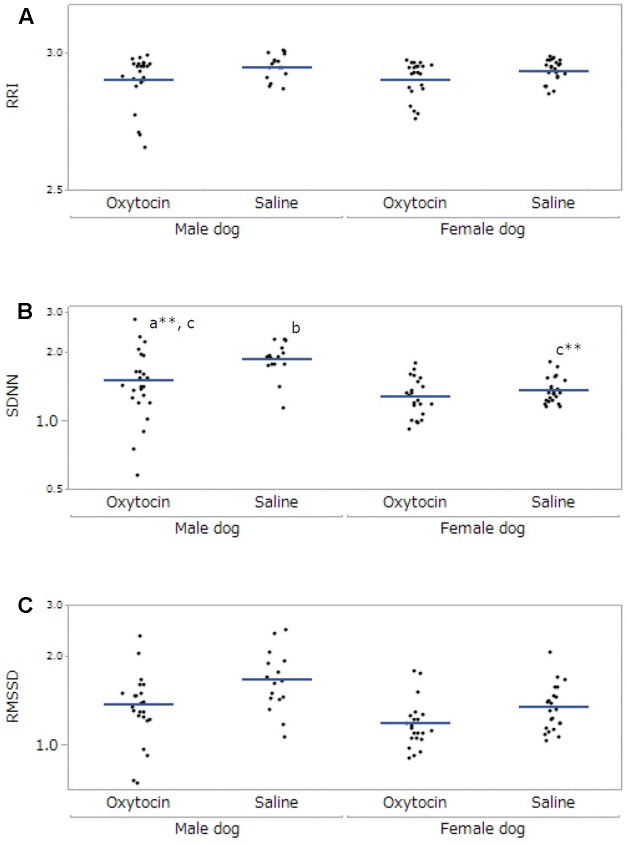
Comparisons of heart rate variability of owners between oxytocin and saline treatment conditions. All parameters, RRI **(A)**, SDNN **(B)**, and RMSSD **(C)**, showed significant differences between oxytocin and saline treatments conditions. Significant dog-sex differences were observed in SDNN and RMSSD. A significant interaction between administration and dog’s sex was found in SDNN. Results are shown with logarithmic. The blue lines indicates the mean of HRV. ^∗∗^*p* < 0.001.

After excluding participants not meeting the inclusion criteria, 12 owners (five male, seven female) and 7 dogs (three male, four female) were included in the final HRV analysis. The data collected from the dogs was not sufficient for statistical analysis; therefore, only the owners’ data were analyzed. A significant main effect was found in administration in RRI (*F*[1,53.99] = 14.10, *p* < 0.001), and the RRI under the oxytocin condition was lower than that under the saline condition (*p* < 0.001, **Figure 3A**). For SDNN, there were significant main effects of dog’s sex (*F*[1,21.68] = 11.71, *p* = 0.002) and administration (*F*[1,55.04] = 33.55, *p* < 0.001), and a significant interaction between dog’s sex and administration (*F*[1,55.04] = 21.29, *p* < 0.001). *Post hoc* tests indicated that the SDNN of the owners of male dogs was higher than that of the owners of female dogs (*p* = 0.002), and the SDNN under the oxytocin condition was lower than that of the saline condition (*p* < 0.001). Moreover, the SDNN of the owners of male dogs under the oxytocin condition was lower than that under the saline condition (*p* < 0.001), and owners of male dogs showed a higher SDNN than owners of female dogs under the saline condition (*p* < 0.001, **Figure 3B**). In RMSSD, with significant main effects found in dog’s sex (*F*[1,21.08] = 8.70, *p* = 0.008) and administration (*F*[1,58.35] = 18.93, *p* < 0.001). The RMSSD of the owners of male dogs was higher than that of the owners of female dogs (*p* = 0.008), and the RMSSD under the oxytocin condition was lower than that under the saline condition (*p* < 0.001, **Figure 3C**). No significant main effect of “time of data collection” was found in any HRV parameter.

## Discussion

The aim of the present study was to investigate whether relationships based on the oxycitocinergic positive loop (discovered in European dog breeds) are also built between humans and ancient breeds, which are considered genetically closer to wolves. The results of this experiment demonstrated that, during a 30-min interaction, intranasal oxytocin did not affect the total amount of time during which dogs touched or were at close proximity to participants. However, there was an increase in the duration of the dog’s gazing behavior at its owner. While Nagasawa et al. (2015) reported that intranasal oxytocin only intensified the gazing behavior of female dogs, we observed no difference between the sexes in the present study. It is also known that inter-species differences in oxytocin receptor exist (Donaldson and Young, 2008), as well as sex difference (Rilling et al., 2014). Moreover, the frequencies of the single-nucleotide polymorphisms of the oxytocin receptor gene are significantly different not only among wolves and dogs, but also among different dog breed groups (Tonoike et al., 2016). Therefore, these results suggested that sex differences may exist depending on the dog breeds, such as between European breeds and the more ancient Japanese breeds.

The urinary oxytocin in female dogs increased after 30 min interaction with owner in the current study as well as their owners, and this was not consistent with the results of Nagasawa et al. (2015) in which elevated urinary oxytocin after interaction under oxytocin treatment conditions only occurred in the owners of female dogs, but not in dogs themselves. The enhanced female dog gazing behavior due to oxytocin treatment activated the owner’s oxytocin nervous system. However, since active contact between the owner and the dog was restricted during the interaction, we assume that the positive loop was interrupted and the dogs’ urinary oxytocin was not elevated by 30-min experiment in the previous study (Nagasawa et al., 2015). However, the results of the present experiment suggested that the oxytocin positive loop was not inhibited in this ancient breed. The discrepancies between the current findings and those in the previous study included remarkably fewer approaches made by the dogs to their owners. The mean gaze duration of oxytocin-treated female Japanese dogs was slightly less than 80% of the previous study in European breeds only (Nagasawa et al., 2015: 244.55 ± 63.95 s, this experiment: 190.14 ± 56.60 s), and the mean skin-contact time was only 5.14 ± 2.41 s, and 1/3 of the female dogs did not touch their owners at all (Nagasawa et al., 2015: the mean skin-contact time was 78.78 ± 37.62 s, all oxytocin-treated female dogs touched their owner during 30-min interaction). While time spent in proximity to owners was significantly longer than to unfamiliar persons, it was still at only around 60% of the time recorded in the previous study (Nagasawa et al., 2015: 1011.60 ± 126.77 s, this experiment: 637.91 ± 136.05 s) (**Table 1**). This result is consistent with findings in Konno et al. (2016), which demonstrated that the gaze of the Japanese dog breed Akita was shorter than European breeds, and in Tonoike et al. (2015), which demonstrated that Japanese dog breeds followed and sought out contact with their owners much less frequently than European breeds. Moreover, the result of multiple regression analysis indicated that the elevation rate of urinary oxytocin in the owners decreased when the touch duration with owners was longer, as well as when dogs were males. Takeuchi and Mori (2006) conducted a questionnaire survey of veterinarians, and found that Japanese breeds were scored lower for playfulness and affection demand, and higher for aggression to dogs, watchdogs barking, territorial defense, and snapping at children than European breeds. Based on these characteristics, Japanese dog breeds as pet dogs would normally have less prolonged skin-contact with their owners than European breeds. Therefore, the restricted contact made by the owner during the present experiment is thought to have had no marked effect on oxytocin secretion in the Japanese dogs compared with the European breeds. However, the female dog’s pre-treatment urinary oxytocin level under oxytocin treatment conditions was lower than that under saline treatment conditions, albeit not significantly different. Since the order of administration condition were counterbalanced, thus, we cannot explain this low pre-treatment urinary oxytocin level in female dogs. Therefore, it is essential to consider carefully the elevated urinary oxytocin in female dogs due to treatment with oxytocin in this experiment.

**Table 1:** The comparison between the previous studies and the current study.

(A) The results of oxytocin administration (inhibition of human’s behavior)							
	Gaze	Touch	Proximity	Urinary oxytocin		Heart rate variability		
				Dogs	Owners	RRI	SDNN	RMSSD
European breeds ^2^	F increased (OT)	N.S.	N.S.	N.S.	OW of F increased (OT)	–	–	–
Japanese breeds ^3^	Both M and F increased (OT)	N.S.	N.S.	F increased (OT)	OW of F increased (OT)	OT > S	OT < S (Total and S), M > F	OT < S, M > F

**(B) Dog’s behavior in oxytocin administration (sec/30 min, inhibition of human’s behavior)**			
	**Gaze**	**Touch**	**Proximity**					

European breeds ^2^	244.55 ± 63.95	5.14 ± 2.41	1011.60 ± 126.77					
Japanese breeds ^3^	190.14 ± 56.60	78.78 ± 37.62	637.91 ± 136.05					

**(C) Dog and wolf’s behavior in free-interaction with owner (sec/5 min)**			
	**Dog’s gaze to owners**	**Talking to dogs**	**Touching dogs**

European breeds ^1^	57.93 ± 9.87	28.66 ± 6.34	54.51 ± 13.42
Wolf ^1^	0.21 ± 0.10	22.72 ± 5.36	33.86 ± 18.25

*OT, in oxytocin administration; S, in saline administration; M, male dogs; F, female dogs; OW, owners*.

*^1^Experiment 1 in Nagasawa et al., 2015; ^2^Experiment 2 in Nagasawa et al., 2015; ^3^The current study.*

On the other hand, despite the absence of sex effects on the duration of dogs’ gaze through administration of oxytocin, we observed an increase in urinary oxytocin concentration only in female dogs and their owners. Nagaswa et al. (2009) found a significant positive correlation between the number of behavioral exchanges that were initiated by dog’s gazing behavior at its owner and the increase in the owner’s urinary oxytocin. Therefore, although gaze plays an extremely important role as a trigger in the interaction between dogs and humans, there also needs to be other elements present, or elements that substitute gaze. In this experiment, we did not observe an effect of the sex of the owner on urinary oxytocin levels, but there may be human factors, which was subtle but different between the owners of the male and female dogs, that are involved in the elevation of urinary oxytocin levels.

This experiment was conducted based on the hypothesis that Japanese dogs are intermediates between wolves and European dog breeds concerning their bonding phenotype with humans. While wolves show almost no mutual gaze behavior with their owners, they demonstrate prolonged skin-contact. Furthermore, in previous studies (Nagasawa et al., 2015), the urinary oxytocin levels were significantly higher in wolves than dogs. This can be explained by the high degree of unity and cooperative behavior in wolf packs and their wariness of outgroups. Generally, Japanese dogs are also strongly wary and aggressive toward external parties and are not easily sociable with unfamiliar persons. Therefore, it was hypothesized that their urinary oxytocin levels would be higher than European breeds. Nevertheless, Japanese dogs did not actively touch their owners, unlike wolves, and the urinary oxytocin levels were similar to European breeds. There is a hypothesis that domestication in dogs is related to a modified stress response (Hare and Tomasello, 2005), which is regulated by glucocorticoids. Oxytocin itself antagonizes glucocorticoid secretion (Kikusui et al., 2006); therefore, these two neuroendocrine systems can be modified during the domestication process, either dependently or independently. Based on the results of the current experiment, urinary oxytocin concentrations in Japanese breeds are similar to those of European breeds and lower than that of wolves, suggesting their endocrine systems may differ substantially from that of wolves. The promoter regions of behavior-related genes were differently methylated between wolves and dogs (Banlaki et al., 2017). Investigating the differences in oxytocin, glucocorticoids, and these receptor genes, and epigenetic modifications between Japanese dog breeds and wolves is expected to provide major elucidation of the domestication process.

We also investigated the changes that occur in the autonomic nervous systems of owners during owner-dog mutual gaze. The owners’ RRIs were significantly lower when the dogs were treated with oxytocin compared with saline treatment conditions, which indicated an elevation of heart rate. SDNN, which is an index of the activity of the autonomic nervous system, was also significantly lower when dogs were under oxytocin treatment conditions compared with saline treatment, which indicates that the activity of the autonomic nervous system was decreased. RMSSD, which is an index of the activity of the parasympathetic nervous system, was also lower under oxytocin treatment conditions compared with saline treatment conditions. This indicates that the parasympathetic nervous system activity decreased. It was reported that a decrease in SDNN is related to the stress conditions when in parallel with decreased RMSSD (Task Force of The European Society of Cardiology and The North American Society of Pacing and Electrophysiology, 1996), whereas some studies showed that increase of the parameters of HRV was related to positive emotional states (Kreibig, 2010). The results of the current study correspond more to the former. In human studies, oxytocin promotes reactivity to attachment signals emitted from infants (Riem et al., 2011); thus, interaction with oxytocin-treated dogs enhanced owner’s tension/arousal, probably due to the increase in eye-gaze attachment behavior. There were also sex differences in SDNN and RMSSD; the owners of female dogs displayed lower SDNN and RMSSD. Therefore, the owners of female dogs may have experienced more tension than the owners of male dogs. Further studies are needed to clarify the correlation between owners’ HRV and oxytocin release. In addition, although gaze plays an extremely important role in the interaction between dogs and humans, it is possible there are elements other than gaze affecting the interaction. These yet uncharacterized signals may be modulating the owners’ HRVs differentially for male and female dogs. As discussed above, the differences between the current findings and those from a previous study included remarkably fewer approaches made by the Japanese dogs toward their owners; the mean skin-contact time was only 5.14 ± 2.41 s, and 1/3 of the female dogs did not touch their owners at all (Nagasawa et al., 2015: 78.78 ± 37.62 s). Tonoike et al. (2015) also demonstrated that Japanese dog breeds followed and sought out contact with their owners much less frequently than European breeds. Moreover, the result of multiple regression analysis indicated that the elevation rate of urinary oxytocin in the owners decreased when the touch duration was longer. Based on these results, which differ from European breeds, Japanese dog breeds may use different subtle types of signals for establishing bonds with their owners. Future studies are needed to clarify this issue.

The results that Japanese dogs treated with oxytocin changed their gazing behavior toward their owners, which promoted the secretion of oxytocin in the owners, indicated that Japanese breeds, which were included in ancient breeds, also have the possibility to establish the oxytocin-gaze positive loop with humans; however, their manner of affiliative interaction with humans differed from both European breeds and wolves. Moreover, the interactions with dogs treated with oxytocin may cause the change of the owners’ autonomic nervous system depending on the dog’s sex although the direct effect of mutual gaze with dogs was not found. Considering the difficulty in collecting urine and monitoring their HRV with an electrocardiograph in Japanese breeds and how the mutual gaze between Japanese breeds and their owners was limited, any further discussion on this topic is difficult. In addition, the social experience and learning also modulate the human-directed behavior (Barber et al., 2016; D’Aniello and Scandurra, 2016). Therefore, further detailed analysis, including the factors such as breed, sex, social experience, and age, is needed using a larger dog sample size and direct comparisons with European breeds. These findings pave the way for future studies investigating the possibility of whether ancient breeds, including Japanese dog breeds, adopt different attachment strategies than European breeds.

## Author Contributions

As the first author, MN was involved in all steps of the process, and was the primary writer of the text. MO has been involved data collection and analysis. KM has been involved in the hormone assay, as well as the write up of the text. As supervisor, TK has been involved in the design and has contributed to the write-up.

## Conflict of Interest Statement

The authors declare that the research was conducted in the absence of any commercial or financial relationships that could be construed as a potential conflict of interest.
